# A framework for quantifying geodiversity at the local scale: a case study from the Rokua UNESCO Global Geopark

**DOI:** 10.1098/rsta.2023.0059

**Published:** 2024-04-01

**Authors:** Helena Tukiainen, Tuija Maliniemi, José Brilha, Janne Alahuhta, Jan Hjort

**Affiliations:** ^1^ Geography Research Unit, University of Oulu, P.O. Box 3000, FI-90014 University of Oulu, Finland; ^2^ Institute of Earth Sciences, Pole of the University of Minho, Campus de Gualtar, Braga, Portugal

**Keywords:** beta diversity, geoconservation, geodiversity, geopark, landform, topographical heterogeneity

## Abstract

Geoconservation and related quantitative and qualitative geodiversity assessments are gaining increasing attention. However, methodologies for measuring geodiversity at local scale are currently rare. Here, we present a framework for assessing local-scale geodiversity of different landforms using field-based and digital elevation model (DEM-) derived data from the Rokua UNESCO Global Geopark in Finland. We observed the presence or absence of various geodiversity elements, such as geological or topographical elements in our study sites, and used these data to quantify alpha (α), gamma (γ) and beta (β) geodiversity of various landforms. In addition, we measured topographical heterogeneity in the field and from DEMs. The results showed distinct patterns in the geodiversity and topographical variation of the landforms. The differences between α, γ and β geodiversity of different landforms were particularly clear. According to the results, measures of topographical variability can be used to some extent as surrogates for geodiversity, but the choice of optimal variables is context and scale dependent. These results provide perspectives for further local-scale geodiversity assessments in different study areas and are applicable for a range of purposes, from scientific research to practical management and geoconservation.

This article is part of the Theo Murphy meeting issue ‘Geodiversity for science and society’.

## 1. Introduction

Geodiversity is being increasingly integrated into nature conservation efforts at different spatial scales. These efforts range from global initiatives such as the United Nations Educational, Scientific and Cultural Organization (UNESCO) Global Geoparks and World Heritage to local geosite assessments and geodiversity action plans [1–3]. To complement the existing approaches to assessing geodiversity at the local scale, there is a need for methods that combine information on both qualitative geodiversity (e.g. which geodiversity elements, such as rock types, are present at the site) and quantitative geodiversity (e.g. number of different types of geodiversity elements are present at each site) ([4–7]; table 1). An integrated approach to assess geodiversity would both support geoconservation and increase our knowledge of geodiversity patterns [14]. For example, defining quantifiable geoindicators would be important for the monitoring of sites with high geoheritage value under human presence, such as International Union of Geological Sciences (IUGS) geological heritage sites [15–17].

A framework for quantifying geodiversity through the concepts of alpha (α), beta (β) and gamma (γ) geodiversity has recently been presented (table 1; [18]). In particular, it highlights the importance of recognizing the composition of geodiversity elements, or geocomposition, between different sites (i.e. β geodiversity). Both α and γ geodiversity can be measured e.g. with simple richness measures or diversity indices, whereas β geodiversity can be calculated with different distance-based statistical methods [18–21]. To date, most quantitative geodiversity assessments have been carried out at the α geodiversity level, i.e. assessing the number or abundance of geodiversity elements at a site, or sometimes at the γ geodiversity level, which represents the total diversity of geodiversity elements in a region [18,22]. For example, different types of quantitative geodiversity maps typically present geodiversity information at the α or γ level (e.g. [23–25]). The β geodiversity approach, in turn, quantifies the difference between study sites in terms of their geocomposition but has not been widely applied so far. A recent example is provided by Erikstad *et al.* [20], who used principal component analysis ordination to study landscape-scale β geodiversity in Sweden and Norway. However, they lacked landform data, which is an important aspect of geodiversity.

**Table 1.** Key concepts of this study. In addition to a short explanation of each concept, key references for more detailed information on each concept are listed.

concept	explanation	key reference
Alpha (α) geodiversity	The number or abundance of geodiversity elements at a site. For example, the number of different types of geodiversity elements in one site is a measure of α geodiversity.	[8]
Beta (β) geodiversity	The variation of geodiversity elements (or geocomposition) among sites.	[8]
Digital Elevation Model (DEM)	A geospatial (GIS-based) representation of the elevation data of an area. In this study, we use grid-based DEMs at 2 m resolution.	[9]
Gamma (γ) geodiversity	The number of different geodiversity elements in a region or across all study sites/units.	[8]
Geocomposition	The composition of geodiversity elements in a site. For instance, a site includes fluvial erosion, fluvial deposition and slow mass movements, whereas the other includes organic deposits and organic hummocks. These two sites have completely different geocomposition.	[8,10]
Geodiversity element	Geological, geomorphological, hydrological and topographical elements that together form the geodiversity of the site. Synonym for a feature of geodiversity i.e. geofeature. In electronic supplementary material S1 there is a comprehensive list of geodiversity elements observed in our study sites.	[11]
Geomorphon	A topographical element that contributes to the local geodiversity and sets the main characteristic of a certain terrain. They are classified into ten different terrain forms: flat, peak/summit, ridge, shoulder, spur, slope, hollow, footslope, valley and pit/depression.	[12]; table 2
Georichness	The number of different types of geodiversity elements observed in a study site. For example, if there are ten organic hummocks and one fluvial deposition in a site, the georichness value of that site is 2. Can be used as a measure of α geodiversity.	[11]
Qualitative geodiversity information	Description of the different geodiversity elements in a site. For instance, the type of geodiversity element, or the type of landform where the study site is located (as in this study), is qualitative information on geodiversity.	[13]
Quantitative geodiversity information	Numerical assessment of the number of different geodiversity elements in a site (geodiversity index). For example, georichness is a quantitative measure of geodiversity.	[13]

Although a variety of quantitative methods for measuring geodiversity have been proposed, only a handful of them are targeted for local scale assessments [22,26]. Field work can be used as a direct method to gain geodiversity information of an area, whereas indirect methods using a geographic information system (GIS) are often used as a time and money saving way for geodiversity calculations [13]. Recently, a cost-efficient field method for measuring local-scale geodiversity has been introduced by Hjort *et al.* [11]. There, the assessment of geodiversity is based on observing the presence of geological, hydrological and geomorphological geodiversity elements at the study sites on a scale of a few to tens of metres. This method provides information on the geodiversity elements of each study site, which can be further used to quantify α, β or γ geodiversity [18]. One indirect way to assess geodiversity at the local scale has been to use digital elevation model (DEM) based measures (e.g. [9,27,28]), derived from high-resolution DEMs. However, it has been stated that topographical measures alone do not provide a good overall picture of the geodiversity of an area, as they only indicate one aspect of geodiversity [29].

In addition to quantitative geodiversity assessments, there is a need for qualitative approaches. While quantitative methods are derived from field measurements, numerical calculations, or GIS analysis of raw data [13], qualitative assessments are usually descriptive and based on expert knowledge. As an example of a qualitative approach, Tukiainen *et al.* [30] examined the plant species diversity on different landforms (considered as qualitative units of geodiversity) in the Rokua UNESCO Global Geopark in Finland. In addition to purely quantitative or qualitative approaches, there are also examples of recent landscape-scale studies that cover both quantitative and qualitative aspects of geodiversity (e.g. [6,7,31,32]). For instance, in Brazil, Gonçalves *et al.* [32] investigated how geodiversity units (qualitative data) correlated with the location of sites of geological interest, and with other aspects of the environment, such as land use and vegetation. However, local-scale assessments combining both qualitative and quantitative aspects of geodiversity remain rare (but see [33], where an omnidiversity approach was taken, where information on geodiversity and biodiversity were combined at geoconservation sites in Tasmania).

**Figure 1.**
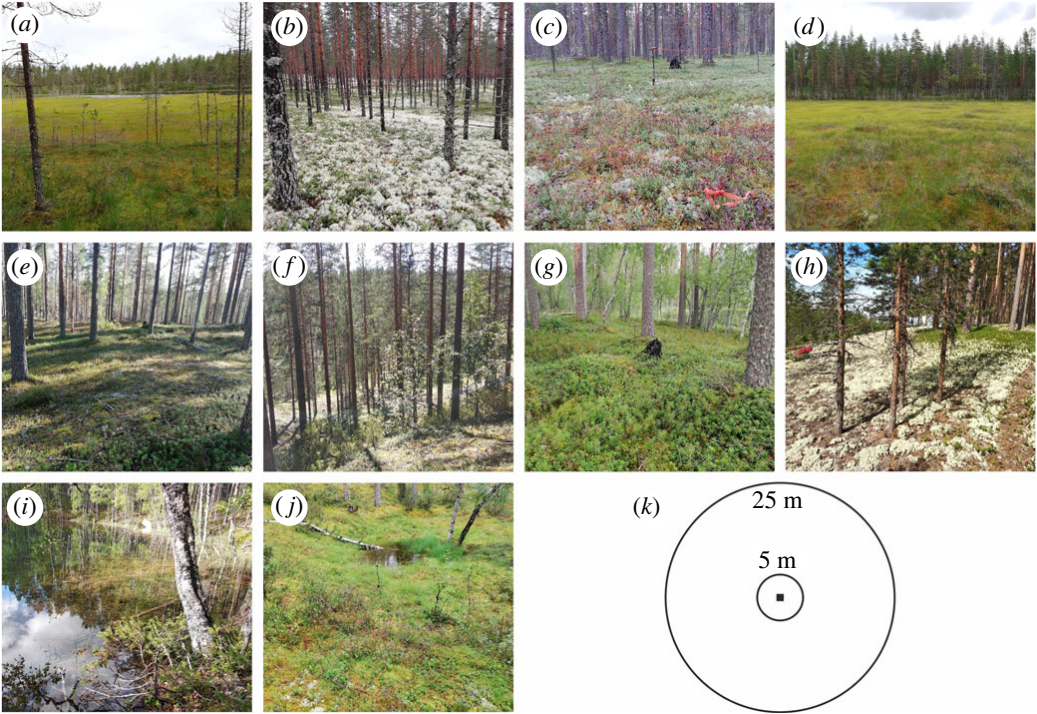
Example photos of the studied landforms: (*a*) level aapa mire (i.e. mire without hummocky surface topography), (*b*) aeolian deposit, (*c*) glaciofluvial-aeolian plain, (*d*) hummocky mire, (*e*) kame, (*f*) kettle hole, (*g*) peat hummocks, (*h*) sand dune, (*i*) shore and (*j*) temporary pond. In (*k*) there is an illustration of the study set-up with the two circles of different radius size (5 and 25 m) which were placed in each of the study sites (*n* = 43). Photos: Jan Hjort (*a*–*e*, *g*, *j*), Helena Tukiainen (*f*, *h*, *i*). (Online version in colour.)

In this study, we present a framework for assessing local-scale geodiversity from field-based and DEM-derived data. We use data from 43 study sites that located on qualitative study units, i.e. on different landforms, such as sand dunes, lake shores and temporary ponds (figure 1). The study sites were circles of 5 and 25 m radius (with the same centre location; figure 1*k*) in Rokua UNESCO Global Geopark in Finland. In the field, we observed the presence or absence of different geodiversity elements (geological, geomorphological, hydrological and topographical elements; [34]) within each circle using the field method of Hjort *et al.* [11]. We adapted the original method by adding observations of topographical elements (based on geomorphons; cf. [35]). In addition to these geodiversity data, we calculated several field-based and DEM-derived measures of topographical heterogeneity for our study sites. Our main aim is to demonstrate, how local-scale geodiversity can be quantitatively measured between different qualitative study units (landforms). Specifically, we investigate (i) how local-scale geodiversity of landforms can be quantified at α, β and γ levels, and (ii) how the size of the study unit (5 or 25 m radius circle) affects the assessment of geodiversity. In addition, we analyse how local-scale geodiversity correlates with measures of topographical variation (estimated in the field and calculated using a DEM) in our study sites. In this study, α geodiversity is the number of different geodiversity elements on each landform and γ geodiversity is the sum of all geodiversity elements across all the sites located on the same type of landform. Beta geodiversity is variation among geodiversity elements among sites and is measured as the Jaccard dissimilarity index (table 1).

## 2. Material and methods

### (a) Study area

**Figure 2.**
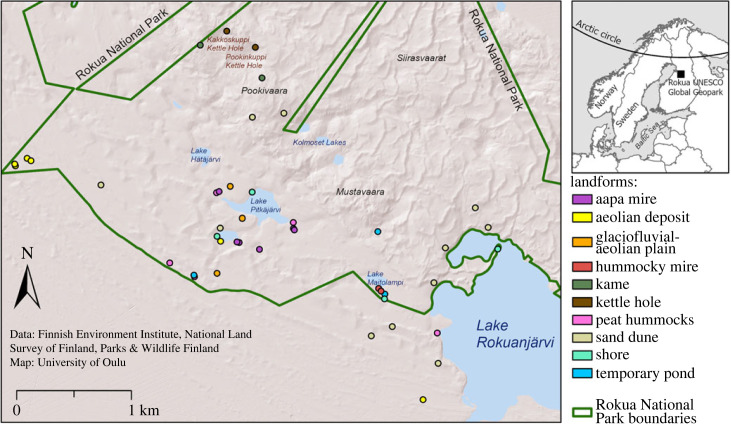
Map of the study area in Rokua UNESCO Global Geopark and location of the study sites on different landforms. The background is a hillshade surface model derived from a 2 m resolution DEM (altitude angle of the light source = 45°, azimuth = 315°) [36]. (Online version in colour.)

The study sites are located inside or in the vicinity of Rokua National Park in Finland (64.56° N, 26.49° E), which is also protected under the European Union's Natura 2000 network (figure 2). The sites are also located within the Rokua UNESCO Global Geopark. The national park was established in 1956, and the geopark in 2010; both are based on the distinctive geological formations of the last Ice Age. Finland's national parks are managed by Parks and Wildlife Finland under the International Union for Conservation of Nature protected area category II, which means that they are large natural or near natural-state areas that protect large-scale ecological processes with characteristic species and ecosystems [37]. Rokua National Park is one of the smallest national parks in Finland (17 km^2^), with approximately 20 000 visitors per year. It is the most important protected area for Cladina-type/barren heath forests in Finland [38].

Our study sites are in the Rokua esker and dune area, or Rokuanvaara area, which is one of the main landscape areas of the geopark. The theme of the geopark is the heritage of the Ice Age, and the Rokuanvaara area is characterized by the traces of the last continental glacier and its retreat - lichen-covered dunes, kettle holes and crystal-clear kettle hole lakes (figure 1). The bedrock of the area is mostly granite-gneiss-migmatite complex, schists and granite, covered by unconsolidated sediments mostly consisting of glaciofluvial deposits such as sand or silt [39–41]. The highest point of the study area is Pookivaara hill which rises 194 m above sea level and approximately 50 m above the surrounding area.

The study area belongs to the middle boreal vegetation zone [38,42]. The climatic conditions are continental, and the mean annual temperature for the period 1991–2021 was 2.5°C. The mean annual precipitation for the same period was 600 mm. Snow is usually present in the area from October to April [43]. The last known large forest fire in the area occurred in 1860, when almost the entire area burned down [38]. Groundwater level fluctuation is typical, but a general slowly declining trend has been observed in the recent decades [44]. The lake and river system of the area consists of Lake Rokuanjärvi and numerous kettle-hole lakes distributed throughout the park (figure 2).

### (b) Qualitative assessment: landforms

The locations of the study sites are based on a vegetation resurvey carried out in 2021, where 43 sites were relocated based on an old vegetation inventory by Jalas [45]. Observation sites located on ten different landforms (figures 1 and 2): (a) (level) aapa mires: boreal mire complexes with flarks and peat strings, without hummocks; (b) aeolian deposits: flat, wind-deposited sand landforms (e.g. without ridges); (c) glaciofluvial-aeolian plains: topographically flat terrains with a (thin) layer of wind-deposited sand underlain by glacial meltwater accumulations; (d) hummocky mires: aapa mires with a hummocky topography (diameter of flat-topped hummocks >1 m); (e) kames: hills composed of sand and gravel that are laid down by glacial meltwater; (f) kettle holes: usually circular depressions formed by melting of a block of (buried) glacier ice; (g) peat hummocks: areas of peat or peat-covered Earth hummocks (10–50 cm high, <1.5 m in diameter); (h) sand dunes: distinct ridges formed by windblown sand; (i) shores: marginal zones above the mean water level of a lake (horizontally <5 m from the waterline); and (j) ephemeral ponds: temporary ponds or pools (e.g. seasonal flood ponds).

### (c) Quantitative geodiversity methodology

Our quantitative geodiversity data are based on a recently introduced field method by Hjort *et al.* [11] and were collected in the study area in August 2021. The basis of the field method is in determining the presence of different geodiversity elements in a study site. Here, we made these observations at 43 locations (figure 2) which correspond to the locations of the vegetation inventory. As Hjort *et al.* [11] state, it is important to adjust the field method based on local environmental conditions. We adapted the geodiversity element classification for boreal environments by focusing on those elements found from this area (e.g. excluded the cryogenic elements that were in the original [11] classification) and decided to make the observations from circles with 5 and 25 m radius (figure 1*k*). The fieldwork in this study was conducted by a geomorphology expert, but the field method is designed to be user-friendly and simple enough for a non-geomorphologist with a reasonable amount of training (e.g. a few days in the field under the supervision of a more experienced geomorphologist) [11], furthermore, the method does not require technical resources. In this study, up to 22 sites were mapped in one field day.

The presence of geological, geomorphological, hydrological and topographical elements were observed in the field at each study site. The full list of observed geodiversity elements and the number of study sites where each of these elements was observed is in electronic supplementary material, S1. Geological elements are determined based on the material itself, and we found two types of geological elements: coarse sediments (sand or gravel) and peat. Geomorphological elements are classified into main process groups (aeolian, fluvial, littoral, organic, mass movements, nivation, anthropogenic, raindrop, glacigenic and glaciofluvial), which are then subdivided into individual elements (electronic supplementary material, S1). For example, from the fluvial elements we identified fluvial erosion and fluvial deposition. In total, we identified 13 geomorphological elements. Hydrological elements are classified according to the type of the water element (spring, ephemeral channel, standing water, dry pond and wetland). To complement the data with topographical elements (which were not included in the original methodology of [11]), we observed the presence of different geomorphons at each study site. These are comparable to the geomorphological forms or geomorphons of Jasiewicz and Stepinski [35], except that our observations were made visually in the field and not from DEMs (see also [46]). Geomorphons are forms of terrain that can be classified into ten different elements: flat, peak/summit, ridge, shoulder, spur, slope, hollow, footslope, valley and pit/depression (tables 1 and 2).

**Table 2.** Topographical elements (geomorphons) that were observed from the study sites. It should be noted that the size limits are scale and study dependent. The illustrations are examples of the elements from a 2 m resolution hillshade surface model [47].

topographical element	description	illustration
flat	relatively flat terrain (>2–3 m^2^;slope <3°)	
		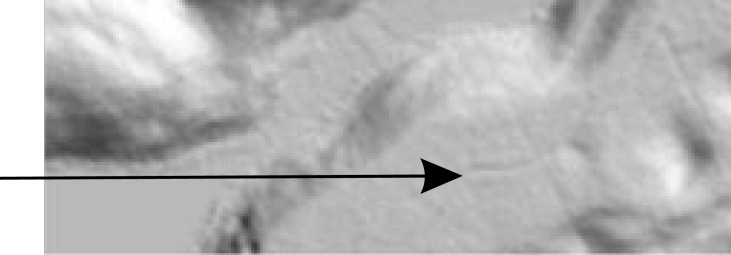
peak	observable mound (>2–3 m^2^; stones/blocks or Earth hummocks are not considered)	
		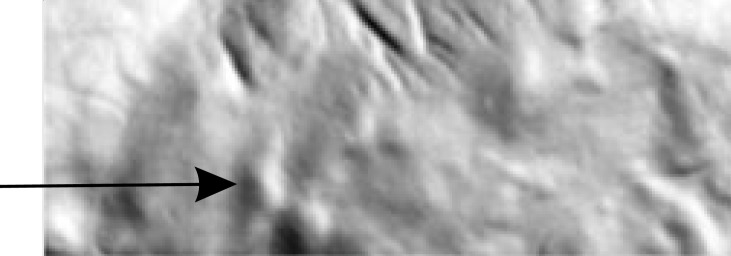
ridge	horizontal, >2–3 m long(elongated) ridge	
		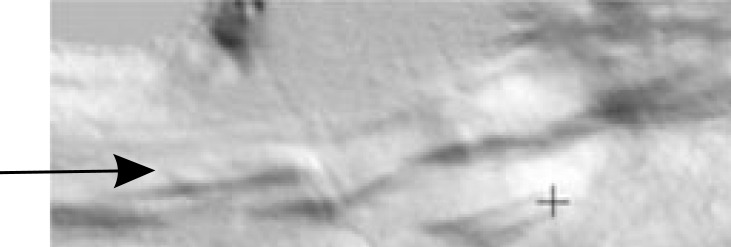
shoulder	an intersection (a bend) of a flat area and a down-slope; >2–3 m long	
		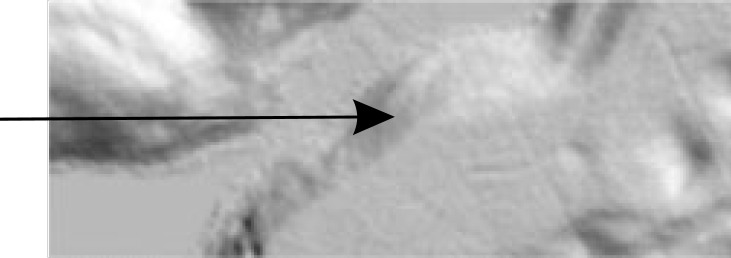
spur	sloping, >2–3 m long(elongated) ridge	
		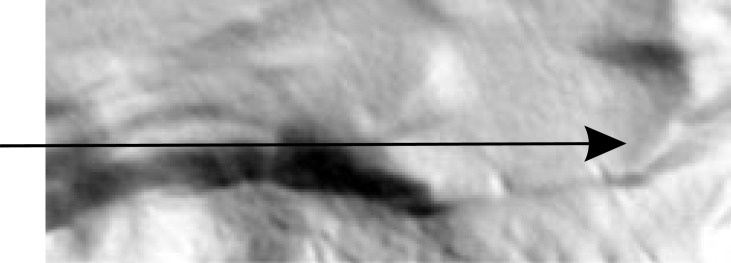
slope	a slope (>5°) area >2–3 m^2^	
		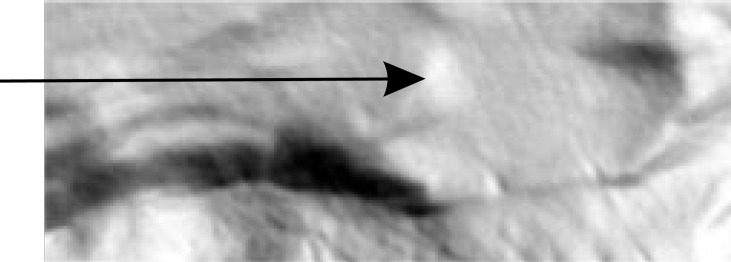
pit	a pit or a depression >1 m^2^	
		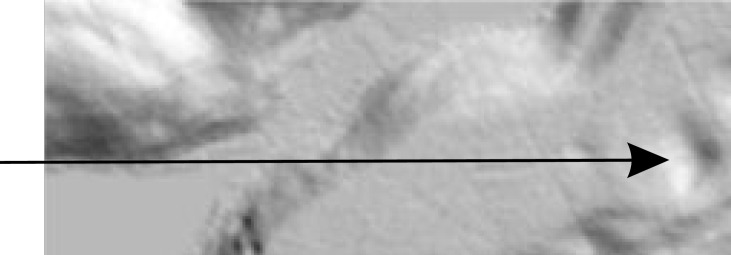
valley	horizontal, >2–3 m long(elongated) depression or channel	
		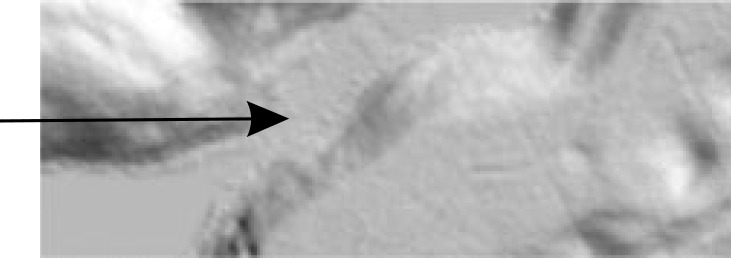
footslope	an intersection (a bend) of an up-slope and a flat area; >2–3 m long	
		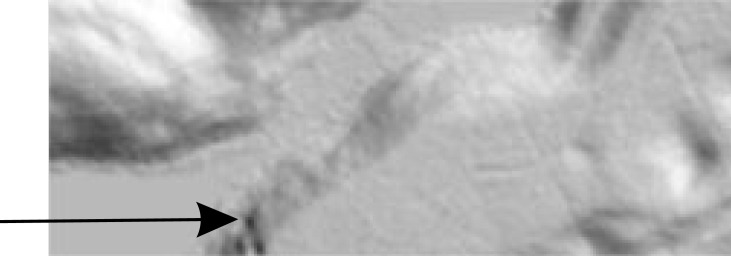
hollow	sloping, >2–3 m long (elongated) depression or channel	
		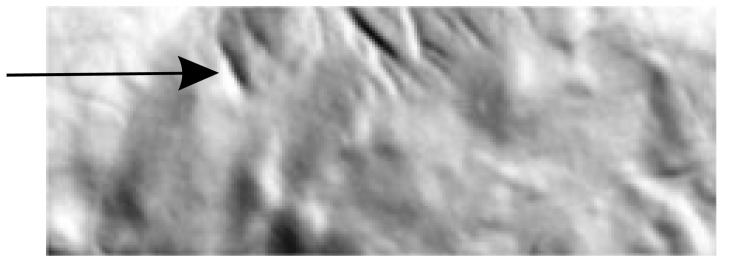

To quantify geodiversity, we followed the framework of α, β and γ geodiversity (table 1; [18]). Alpha geodiversity is the sum of different geological, geomorphological, hydrological and topographical elements of each study circle, and therefore, a synonym for georichness (see e.g. [11,48]). As a measure of α geodiversity, we calculated mean georichness for each landform. Gamma geodiversity, in turn, is the total number of different types of geological, geomorphological, hydrological and topographical elements across all study circles within each landform. These calculations were carried out using Microsoft Excel and R [49], separately for study sites with 5 and 25 m radius. The data, metadata and the R code for the calculations are available in electronic supplementary material, files (electronic supplementary material, S2, S3 and S4).

The site-wise composition of geological, geomorphological, hydrological and topographical elements, i.e. β geodiversity (table 1) was assessed using R by calculating pairwise dissimilarity matrices based on Jaccard dissimilarity coefficients. The matrices were calculated for geodiversity element data using the function *vegdist* from the R package *vegan* [50]. The composition of geodiversity elements (geocomposition) on each landform was visualized using non-metric multidimensional scaling (NMDS) ordination, computed for both matrices using the function *metaMDS* from the *vegan* package. On both NMDSs, the 70% most common and best-fitting different geodiversity elements were added using the *ordiselect* function from the R package *goeveg* [51]. In addition, the Jaccard dissimilarity index was calculated for each landform using the function *β.multi* from the R package *betapart* [52]. This index varies between 0 and 1, where 0 means no difference in geocomposition between sites and 1 means completely different geocomposition between sites.

### (d) Topographical variation data from field surveys

Topographical heterogeneity was observed in the field according to the methodology of Salminen *et al.* [21]. The assessments are based on expert judgement and observation of the topography in the field, by making a detailed survey of each of the 5 and 25 m radius study circles. The assessment value varies between 1 and 10, where 1 indicates very little variation (i.e. nearly flat ground surface) and 10 indicates considerable variation in topography regardless of the slope angle of the site. In electronic supplementary material, S5, there are example photographs of sites that are evaluated with different topographical heterogeneity values. Measurements were made of both micro-scale topographical heterogeneity, which is the variation that occurs on a scale of no more than one metre, and meso-scale topographical heterogeneity, which is the variation that occurs on a scale of more than one metre.

### (e) Topographical variation data from DEMs

We used 2 m resolution light detection and ranging (LiDAR-) based DEMs [36] to calculate DEM-based topographical variables in the studied 5 and 25 radius circles. The approximate elevation accuracy of the DEM was 0.3 m and the point density was at least 0.5 points per square metre [53]. To illustrate the topographical variation of the study sites, the standard deviation of elevation, slope angle [54] and topographical wetness index (TWI; [55]) was calculated for each study circle. These topographical variables correlated well with georichness in a landscape scale study [27]. The calculations were made in ArcGIS Pro 3.1.0 using the *Zonal statistics as table* tool. The relationships between different measures of topographical variation and the georichness of each study circle were analysed using Spearman's rank correlation analysis in the R software [49].

## 3. Results

### (a) Variation of geodiversity on landforms

**Figure 3.**
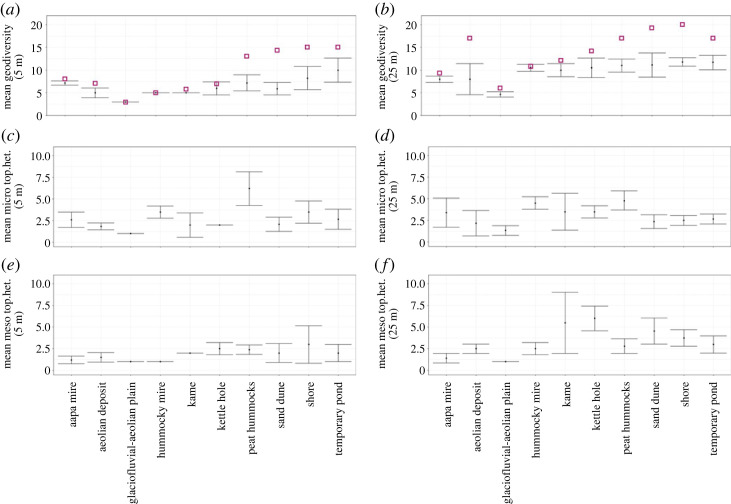
Mean and standard deviation of field-derived local-scale geodiversity (*a*,*b*) and micro-scale (*c,d*) and meso-scale (*e,f*) topographical heterogeneity on different landforms in 5 and 25 m radius study circles. The purple squares represent the total number of geodiversity elements across study circles located on each landform, i.e. γ geodiversity. (Online version in colour.)

According to the results, the α geodiversity (mean georichness) of each landform was close to or slightly lower than its γ geodiversity (total georichness) (table 3 and figure 3). In some cases, such as for sand dunes (*n* = 11), peat hummocks (*n* = 5) and shores (*n* = 4), γ geodiversity was notably higher than α geodiversity. In general, increasing the study circle radius from 5 to 25 m increased α and γ geodiversity of each landform (table 3 and figure 3*a,b*). As an exception, circle size had no notable effect on α or γ geodiversity of aapa mires or temporary ponds. Patterns of β geodiversity (Jaccard dissimilarity index) between study circles of different sizes were somewhat different from α and γ geodiversity patterns (table 3). For example, aapa mires and peat hummocks had a similar sample size (*n* = 5) and similar α geodiversity, but peat hummocks had notably higher γ geodiversity, as well as high β geodiversity, at study sites of both radii (table 3). The β geodiversity of all sites combined (*n* = 43) was very high—close to 1, which means that the geocomposition of our study sites is notably different.

**Table 3.** Local-scale geodiversity and micro- and meso-scale topographical heterogeneity on different landforms and across study sites at 5 and 25 m radius study circles. The highest values among the differently sized circles are bolded for each landform. For α geodiversity and topographical heterogeneity measures, standard deviations are reported after the mean values.

								mean		mean	
		mean		total		Jaccard		topographical		topographical	
		georichness		georichness		dissimilarity		heterogeneity:		heterogeneity:	
		(α geodiversity)		(γ geodiversity)		(β geodiversity)		micro-scale		meso-scale	
landform	sample size	5 m	25 m	5 m	25 m	5 m	25 m	5 m	25 m	5 m	25 m
aapa mire	5	7.2 ± 0.4	**8 **± 0.7	8	**9**	0.13	**0.21**	2.6 ± 0.9	**3.4 **± 1.7	1.2 ± 0.4	**1.4 **± 0.5
aeolian deposit	6	5 ± 1.1	**8 **± 3.4	7	**17**	0.55	**0.71**	1.8 ± 0.4	**2.2 **± 1.5	1.5 ± 0.5	**2.5 **± 0.5
glaciofluvial-aeolian plain	3	3 ± 0	**4.7 **± 0.6	3	**6**	0	**0.43**	1 ± 0	**1.3 **± 0.6	1 ± 0	1 ± 0
hummocky mire	2	5 ± 0	**10.5 **± 0.7	5	**11**	0	**0.09**	3.5 ± 0.7	**4.5 **± 0.7	1 ± 0	**2.5 **± 0.7
kame	2	5 ± 0	**10 **± 1.4	6	**12**	0.33	0.33	2 ± 1.4	**3.5 **± 2.1	2 ± 0	**5.5 **± 3.5
kettle hole	2	6 ± 1.4	**10.5 **± 2.1	7	**14**	0.29	**0.5**	2 ± 0	**3.5 **± 0.7	2.5 ± 0.7	**6 **± 1.4
peat hummocks	5	7.2 ± 1.8	**11 **± 1.4	13	**17**	**0.7**	0.55	**6.2 **± 1.9	4.8 ± 1.1	2.4 ± 0.5	**2.8 **± 0.8
sand dune	11	5.9 ± 1.4	**11.1 **± 2.6	14	**19**	**0.81**	0.76	2.1 ± 0.8	**2.4 **± 0.8	2 ± 1.1	**4.6 **± 1.5
shore	4	8.3 ± 2.5	**11.8 **± 1	15	**20**	**0.71**	0.63	**3.5 **± 1.3	2.5 ± 0.6	3 ± 2.2	**3.8 **± 1
temporary pond	3	10 ± 2.6	**11.7 **± 1.5	15	**17**	**0.55**	0.5	2.7 ± 1.2	2.7 ± 0.6	2 ± 1	**3 **± 1
all sites	43	6.3 ± 2.1	**9.8 **± 2.8	27	**30**	**0.96**	0.95	2.7 ± 1.7	**2.9 **± 1.4	1.9 ± 1	**3.3 **± 1.7

The effect of study circle size was not as pronounced for micro- and meso-scale topographical heterogeneity as it was for α and γ geodiversity (table 3 and figure 3*c–f*). There was slightly more topographical variation in the 25 m radius sites, but especially in the case of micro-scale heterogeneity, there were landforms that gained somewhat similar or higher values in the 5 m radius study circles. For example, peat hummocks (*n* = 5) had substantially high (mean value 6.2 ± 1.9 std) micro-scale topographical heterogeneity in 5 m sites. In general, the trends in the variation of α and γ geodiversity of different landforms, and measures of micro- and meso-scale topographical heterogeneity measures were different from each other (figure 3). There were also similarities: e.g. glaciofluvial-aeolian plains (*n* = 3) were quite low in their geodiversity and micro- and meso-scale topographical heterogeneity, whereas shores (*n* = 4) and temporary ponds (*n* = 3) were characterized by rather high geodiversity and micro- and meso-scale topographical heterogeneity. As a practical example for illustrating the results in detail at a level of an individual study site, we have made a comparison between two study sites that are located in glaciofluvial-aeolian plain (which is a landform with relatively low α geodiversity), and in temporary pond(a landform with relatively high α geodiversity) (electronic supplementary material, S6). There, we have made a detailed list of observed geodiversity elements, α geodiversity and topographical heterogeneity values, and provided example figures from these sites and illustrated the slope angle variation based on 2 m resolution DEM.

**Figure 4.**
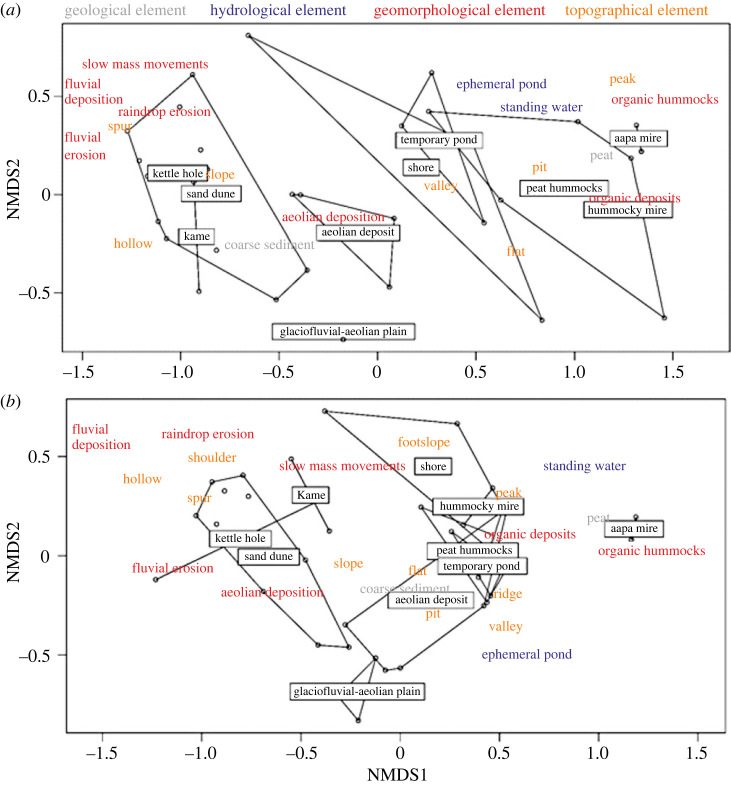
NMDS ordination diagram based on the presence of geodiversity elements and Jaccard dissimilarity for (*a*) 5 m radius study sites and (*b*) 25 m radius study sites. Dots represent study sites that are delimited by different landforms (polygons when *n* ≥ 3 and straight lines when *n* < 3). Labels indicate the location of group centroid in the ordination; 70% of the most common and best-fitting geodiversity elements are shown in the figure. (Online version in colour.)

Ordination made for the 5 m radius study sites showed a clear gradient along the first axis of the NMDS (NMDS1), i.e. a clear difference in the geocomposition (the composition of geodiversity elements) between different landforms (figure 4*a*). Flat aapa mires, hummocky mires and peat hummocks were similar to each other in their geocomposition and clearly different from kettle holes, sand dunes and kames. The geocomposition of glaciofluvial-aeolian planes and aeolian deposits was closer to the latter landforms, whereas shores and temporary ponds were similar to mires and peat hummocks (figure 4*a*). As the radius of the study site increased to 25 m the gradient became less clear, but most of the landforms were still recognizable by their geocomposition (figure 4*b*). The ordination results also showed the most common and best fitting geodiversity elements (figure 4). Kettle holes, sand dunes and kames were characterized by fluvial deposition and erosion, slow mass movements, spurs, hollows and raindrop erosion, whereas more moist landforms (at the other end of NMDS1 axis) were dominated by peaks, peat, standing water and organic hummocks and deposits (figure 4*a*). Both ordinations converged in two dimensions and their stress values were less than 0.2 (0.09 and 0.13 for 5 and 25 m sites, respectively), indicating a good statistical fit [56].

### (b) Correlation among geodiversity and topography

**Figure 5.**
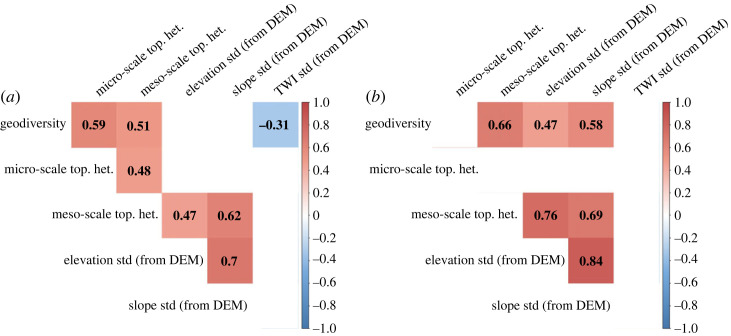
Correlation plot showing the statistically significant (*p* < 0.05) Spearman's rank correlation coefficients of the DEM-based topographical heterogeneity variables and the field-derived topographical heterogeneity and geodiversity values. Correlations are calculated for all (*a*) 5 m and (*b*) 25 m radius study sites (*n* = 43). Red indicates positive correlations and blue negative correlations. Non-significant (*p* ≥ 0.05) correlations are not shown in the graph. (Online version in colour.)

The results of the correlation analysis show some associations between α geodiversity and topographical heterogeneity variables (figure 5; electronic supplementary material, table S2 in Supplementary Material S7). In the 5 m radius study circles, α geodiversity only correlated significantly (*p* < 0.05) with TWI standard deviation (*r*_s_ = −0.31) from the DEM-based topographical variables. There were also significant positive correlations between geodiversity and field-based micro- and meso-scale topographical heterogeneity (figure 5*a*). In the 25 m radius study circles, geodiversity correlated significantly with field-based meso-scale topographical heterogeneity, but also with DEM-based standard deviations of elevation and slope (figure 5*b*). In addition, meso-scale topographical heterogeneity correlated strongly with elevation and slope standard deviation at both study site sizes, whereas micro-scale topographical heterogeneity did not correlate with any DEM-based topographical variables.

## 4. Discussion

In this study, an approach to exploring the variation in local-scale geodiversity that considers both qualitative and quantitative aspects of geodiversity has been presented. Distinct patterns in the geodiversity and topographical variation of different landforms at the α, β, and γ levels have been found. Furthermore, the results highlighted the importance of field-derived information on geodiversity and topographical heterogeneity, compared to DEM-based topographical variables alone. Our results highlight that taking a versatile approach, which is carefully considered for the context of each exploration provides the most useful results in local-scale geodiversity studies.

This study combined qualitative and quantitative geodiversity approaches by calculating the geodiversity of qualitative geodiversity units (landforms). Owing to differences in sample size, it was not meaningful to compare the geodiversity of different landforms. However, the results revealed interesting details about the number of geodiversity elements (α and γ geodiversity) and the occurrence of different geodiversity elements (β geodiversity) on different landforms. The results clearly show that α and γ geodiversity values were higher in the larger study sites (25 m radius). This suggests that, as with species richness [57], georichness tends to increase with the size of the study site (see also [58] where the same trend for landscape-scale geodiversity was observed). Beta geodiversity, in turn, was higher in 5 m radius sites in some landforms, and the gradient in the geocomposition of the landforms was better distinguished in the NMDS ordination analysis performed at the 5 m sites (table 3 and figure 4). Thus, although the 25 m radius circles seems to capture a greater number of geodiversity elements, the 5 m radius circles often seem to capture unique geocomposition. As the circle size increases, more geodiversity elements are potentially located within the circle, which may lead to a homogenization of geocomposition (i.e. lower β geodiversity values). This may be particularly relevant in the case of smaller landforms (such as the temporary ponds in our data; figure 1), where the 25 m radius may extend beyond the landform itself and thus include geodiversity elements outside the actual landform. The best scale for quantifying β geodiversity will therefore depend on the environment and the context in which the assessments are made.

The results of the NMDS ordination analysis (figure 4) highlight the value of landform information in β geodiversity analysis (see [20] where ordination analysis was used in a landscape-scale geodiversity study but lacked landform data). There was a clear pattern in the geocomposition of different landforms: forest-covered landforms with sand and gravel sediments had a very different geocomposition from more moist, peat-dominated landforms. The importance of spatial scale was also evident in the ordination results, as the pattern of difference in geocomposition between the landforms studied was particularly clear at the 5 m radius (NMDS1 axis in figure 4). The ordination results also showed the most common and best fitting geodiversity elements (figure 4). It is especially interesting that topographical elements were characterized differently along the NMDS1 axis: peaks were typical for aapa mires and hummocky mires, whereas spurs, hollows, and slopes were common in kettle holes, sand dunes and kames. This indicates that the inclusion of topographical elements (geomorphons; [35]) was an important addition to the applied field methodology for observing geodiversity [11].

Correlation analysis revealed interesting details of how field-derived α geodiversity, field-derived topographical heterogeneity and DEM-based measures of topographical variation were related (figure 5). According to the results, α geodiversity was positively and significantly correlated with DEM-derived elevation and slope variation at a larger scale (25 m radius circles). This suggests that although topography is only one aspect of geodiversity [59], even fairly simple DEM-based measures may successfully reflect local-scale α geodiversity patterns. This supports the use of different types of DEM-based roughness measures in geodiversity calculations (e.g. [28,60,61]). However, the extent to which purely DEM-based measures can be used to represent the full range of local-scale α geodiversity of study sites should be further tested in different types of environments and study sites of different sizes. One aspect that needs further consideration is the resolution of the DEM (e.g. [62]). For example, in our results field-derived meso-scale topographical heterogeneity (topographical variability at >1 m scale) correlated positively and significantly with DEM-based measures (figure 5). By contrast, micro-scale topographical variation (variability at <1 m scale) had no correlation with DEM-based measures. This probably indicates that the resolution of the used DEM (2 m cell size, accuracy of the elevation 0.3 m; [53]) was not sufficient to observe the fine-scale abiotic variation of the study sites.

In this study, a number of methods have been tested that can be used to estimate the local-scale geodiversity of an area. Although the results are encouraging and provide interesting insights into issues related to scale, diversity levels and the use of field-derived versus DEM-based data, there are issues that should be further developed. For future research, it would be interesting to not only compare measures of geodiversity and topographical heterogeneity between the same sites, but also to compare the geodiversity of different landforms (e.g. to apply and extend the approach by Crisp *et al.* [33], who compared the geodiversity of three single geoconservation sites). With larger sample sizes from each landform (or other qualitative study unit, such as a geological heritage site), it would be possible to obtain information on the statistical significance of these differences. Combining local-scale geodiversity data with vegetation data [42,63] would provide information on the connections between geodiversity and biodiversity in the Rokua area. As the set of landforms analysed was unique to our study area, it would be important to extend further studies of local-scale geodiversity into different environments and locations. For example, local-scale geodiversity studies in morphogenetically different landscapes, such as mountains and plains [64], studies in areas with different human impacts [65], or studies in areas where a different set of geodiversity elements characterize the landscape (e.g. in our study, there were just two geological elements) would be an important extension of our knowledge on local-scale geodiversity patterns.

The approach presented for analysing local-scale geodiversity of landforms has many potential applications to support geodiversity research and geoconservation management. Geoconservation would benefit from an approach that combines qualitative aspects (e.g. the detection of geological heritage sites or landforms) and quantitative aspects (e.g. a thorough examination of the geodiversity of these sites or landforms) [7,33]. For example, in the case of our study, aapa mires (*n* = 5) and aeolian deposits (*n* = 6) had somewhat similar α geodiversity (i.e. they were equally important in terms of mean geodiversity values) (table 3 and figure 3). However, the γ geodiversity of aeolian deposits was high (in the 25 m radius study sites) and the geocomposition of these two landforms were different (figure 4). Thus, although the α geodiversity of aapa mires and aeolian deposits appears to be equal, the γ and especially the β geodiversity analyses point to their unique contribution to the overall geodiversity of the area. Therefore, it is important to consider both landforms in geoconservation management in the Rokua area. Our results also show the value of DEM-derived data in addition to field data: DEMs can be a valuable addition but not the only source of geodiversity information when investigating the local-scale geodiversity of an area [66]. Especially in environments where landforms and geodiversity elements are covered with ground vegetation and tree cover, field work and the combination of different methodological aspects helps in elucidating the landscape and its abiotic characteristics.

This study shows further perspectives for geoconservation actions, as the definition of quantifiable geoindicators, such as numerical geodiversity measures at α, β and γ levels, is very important in the monitoring of geological heritage sites under human presence [3,15,17]. The existence of geoindicators that can measure quantifiable short-term changes in geodiversity (such as the measure of georichness) can make monitoring an effective tool for site management. For example, the presented local-scale geodiversity methodology could be further used to calculate the tourism-carrying capacity of different sites with different types of human presence and use (e.g. the trampling by visitors; [67,68]). In addition, local-scale geodiversity methods could be useful for assessing changes in geological heritage sites caused by weathering and erosion in geological and/or geographical contexts where these processes may be particularly strong (e.g. sites with soft rocks or loose sediments, hot and humid climates, or steep slopes) [69]. In general, the basic procedures for local-scale geodiversity investigations with the framework we have introduced in this study are easy to apply and are time and cost-efficient, although they include field work. The field methodology is designed to be user-friendly and depending on the environment, it is possible to survey ten or more study sites in a day [11]. The following analysis, e.g. calculation of DEM-derived data and the conducting of statistical analysis can take several weeks, depending on the expertise of the analyst and the number of approaches taken. Through different stages, different procedures can be applied and managed, for instance, by using the original field methodology introduction [11], this study and the attached study dataset and R-code (electronic supplementary material, S2–S4) as a base line.

## 5. Conclusion

In this study, we have comprehensively implemented a set of methods for assessing local-scale geodiversity that are applicable to different areas, environments and purposes. We have demonstrated that local-scale geodiversity can be measured quantitatively among different landforms. In particular, we have shown that

(i) geodiversity of landforms can be quantified at α, β, and γ levels,
(ii) the smaller study scale (5 m radius) better revealed the differences in geocomposition of the landforms than the larger scale (25 m radius), and
(iii) measures of topographical variability can be used as surrogates for geodiversity, but the choice of optimal variable(s) is context dependent.

The results therefore highlight the importance of considering the scale of the study and the type of geodiversity information required when conducting geodiversity assessments. When planning a local-scale geodiversity assessment, we recommend considering the following questions on a case-by-case basis:

(i) Is there is a need to assess only the number of geodiversity elements on a site or in a region (α or γ geodiversity), or would information on the geocomposition between different sites (β geodiversity) also be valuable?
(ii) Is there is a need for information at very small scales, or does the study design require larger study units covering tens of metres?
(iii) Is there a need for information only on topographical variation (where DEM-based measures may be sufficient), or more widely on landform and geodiversity patterns (where field work is the basis of the evaluations)?

Answering these questions will pave the way for a local-scale geodiversity framework that can be applied to the specific target of each investigation.

## Data Availability

The data are provided in electronic supplementary material [70].
